# Aquaporins with anion/monocarboxylate permeability: mechanisms, relevance for pathogen–host interactions

**DOI:** 10.3389/fphar.2014.00199

**Published:** 2014-09-01

**Authors:** Janis Rambow, Binghua Wu, Deike Rönfeldt, Eric Beitz

**Affiliations:** Department of Pharmaceutical and Medicinal Chemistry, Christian-Albrechts-University of KielKiel, Germany

**Keywords:** aquaporin, aquaglyceroporin, anion channel, monocarboxylate transporter, formate–nitrite transporter

## Abstract

Classically, aquaporins are divided based on pore selectivity into water specific, orthodox aquaporins and solute-facilitating aquaglyceroporins, which conduct, e.g., glycerol and urea. However, more aquaporin-passing substrates have been identified over the years, such as the gasses ammonia and carbon dioxide or the water-related hydrogen peroxide. It became apparent that not all aquaporins clearly fit into one of only two subfamilies. Furthermore, certain aquaporins from both major subfamilies have been reported to conduct inorganic anions, such as chloride, or monoacids/monocarboxylates, such as lactic acid/lactate. Here, we summarize the findings on aquaporin anion transport, analyze the pore layout of such aquaporins in comparison to prototypical non-selective anion channels, monocarboxylate transporters, and formate–nitrite transporters. Finally, we discuss in which scenarios anion conducting aquaporins may be of physiological relevance.

## INTRODUCTION

**FIGURE 1:**
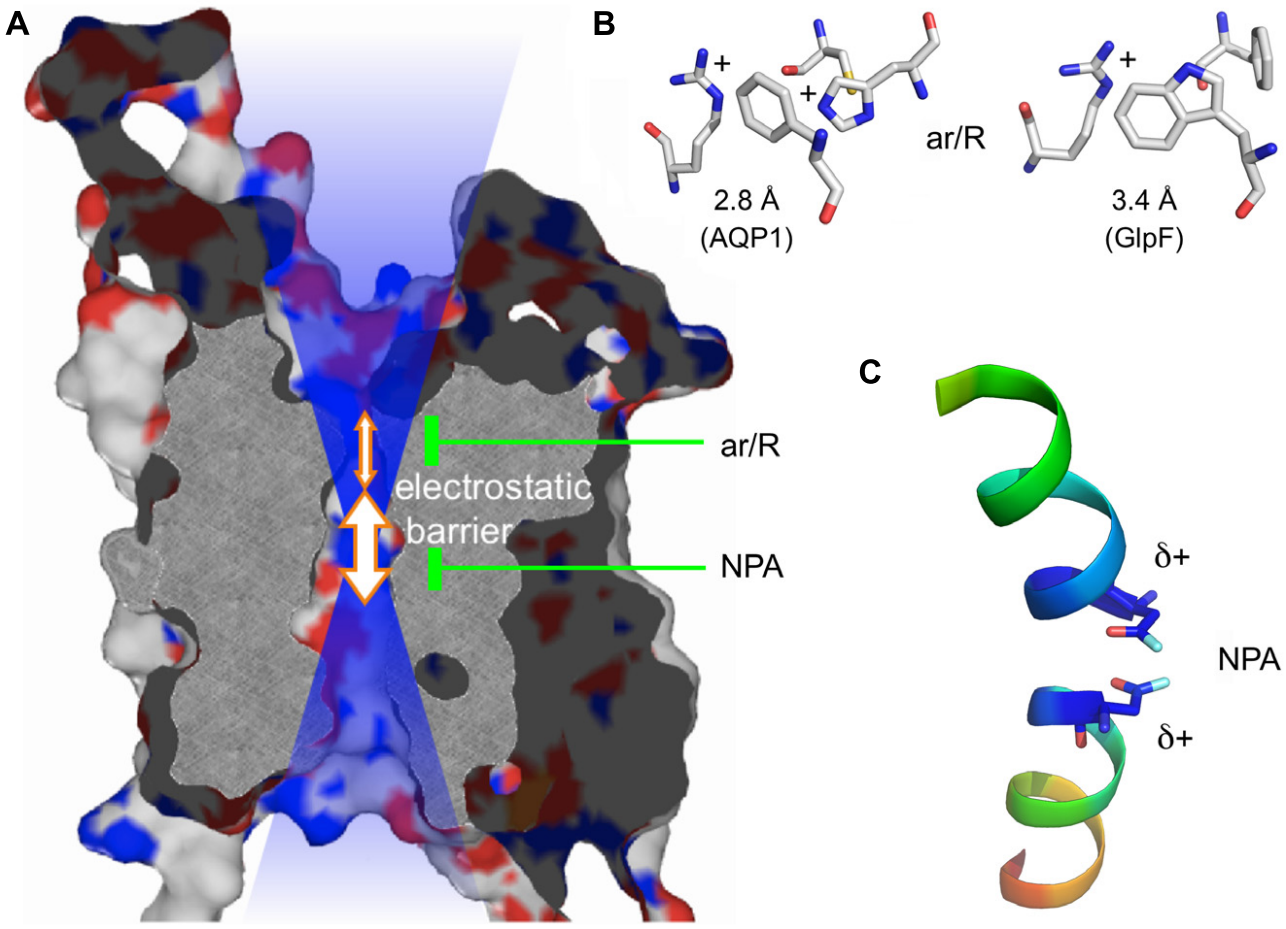
**Protein structure of an AQP monomer. (A)** Slab of AQP1 showing the channel and the two constriction sites. The positive electrostatic field emanating from the channel is symbolized by the blue transparent shading. **(B)** Typical amino acid composition of the ar/R region in a water specific AQP (here AQP1; PDB #1J4N; Borgnia et al., 1999) and an aquaglyceroporin (here *Escherichia coli* GlpF; PDB #1FX8; Fu et al., 2000). **(C)** Half helices B and E with the capping asparagine residues residing at the positive helix ends.

Cellular aquaporin water channels (AQPs) constitute a large family of transmembrane proteins throughout all kingdoms of life (Abascal et al., 2014). AQPs are members of the major intrinsic protein (MIP) family and feature a uniform molecular structure consisting of six transmembrane spans and two half helices that form a seventh pseudo-transmembrane domain (Murata et al., 2000). AQPs are arranged as homotetramers with each monomer contributing an individual transduction channel. The central pore of certain AQP tetramers appears to enable permeation of gaseous substrates (Geyer et al., 2013). Since their discovery about 20 years ago it has become apparent that AQPs conduct a variety of substrates besides water (Wu and Beitz, 2007). Among these are small, uncharged polyols, such as glycerol, other solutes, such as carbonyl compounds (Pavlovic-Djuranovic et al., 2006), urea, or hydrogen peroxide (Bienert et al., 2007; Almasalmeh et al., 2014). Even neutral, protonated arsenous (Liu et al., 2002; Wu et al., 2010) and silicic acid (Ma et al., 2006) have been found to pass. Further substrates of AQPs are solubilized gasses, namely ammonia (Jahn et al., 2004; Zeuthen et al., 2006) and carbon dioxide (Nakhoul et al., 1998). Especially the latter has been linked to permeation through the central pore (Musa-Aziz et al., 2009). Apart from this, AQPs are rather strict about the exclusion of charged substrates. None of the known natural AQPs was found to conduct cations, such as protons (Murata et al., 2000; Tajkhorshid et al., 2002; Ilan et al., 2004; de Groot and Grubmüller, 2005; Beitz et al., 2006b; Li et al., 2011; Wree et al., 2011), inorganic cations (Na^+^, K^+^; Wu et al., 2009), or ammonium (NH_4_^+^; Beitz et al., 2006b; Zeuthen et al., 2006). The case appears somewhat less stringent when it comes to anion permeability of AQPs as discussed in this review. Generally, AQPs have two selective motives. One is located at the extracellular mouth of the pore displaying a highly conserved arginine residue in an aromatic surrounding, the so-called selectivity filter or aromatic arginine (ar/R) region. A second one resides in the center of the channel where the two half helices meet (**Figure 1**; de Groot and Grubmüller, 2001). This second constriction comprises two well-conserved Asn-Pro-Ala triplets (NPA) as capping structures of the half helices with the two Asn residues pointing toward the channel lumen. The diameter of the ar/R region defines selectivity by size exclusion resulting in water specific, orthodox AQPs (<2.8 Å) or glycerol/urea-conducting aquaglyceroporins (>3.4 Å; Fu et al., 2000; Tajkhorshid et al., 2002; de Groot and Grubmüller, 2005; Beitz et al., 2006b). Due to its positive charge, the ar/R region is further involved in proton repulsion (Beitz et al., 2006b; Li et al., 2011). It further forms a joint filter together with the electrostatic field emanating from the positive ends of the half-helix dipoles in the NPA region against the passage of inorganic cations (Wu et al., 2009; Wree et al., 2011; Kosinska Eriksson et al., 2013).

Inversely, positive electrostatics may attract anions to the AQP channel. Yet, the literature holds only one example of a natural AQP, i.e., mammalian AQP6, that is permeable for inorganic anions (Yasui et al., 1999; Hazama et al., 2002; Ikeda et al., 2002; Beitz et al., 2006a; Yasui, 2009). AQP6 is found in the membranes of intracellular vesicles of intercalated cells of the kidney medulla and conducts, among others, nitrate and chloride ions (Yasui et al., 1999). Later, an artificial point mutant of the human water-specific AQP5 was shown to exhibit anion conductance with a permeation sequence equal to AQP6 (Qin and Boron, 2013). Recent studies report that certain AQPs mainly from bacteria and eukaryotic parasites exhibit permeability for weak organic mono-acids, such as lactic acid (Choi and Roberts, 2007; Faghiri et al., 2010; Bienert et al., 2013). Due to the chemical equilibrium, the proportion of deprotonated acid anions vs. protonated, neutral acid molecules depends on the prevailing pH. It favors the anion state at neutral to alkaline conditions and the neutral acid form in the acidic range. Earlier, human AQP9 has been suggested to play a role in the transport of lactic acid and of ketone bodies including the acidic 2-hydroxybutyrate, across the blood–brain barrier (Tsukaguchi et al., 1998, 1999).

In this review, we summarize hypotheses on the molecular mechanisms underlying anion permeability of AQPs as well as implications of monocarboxylate-transporting AQPs for pathogen–host interactions.

## AQPs WITH PERMEABILITY FOR INORGANIC ANIONS

### HUMAN AQP6 IS A NITRATE/HALIDE CHANNEL

The initial biochemical characterization of AQPs in the 1990s led to the view that AQPs are basically passive but selective water or glycerol channels of the plasma membrane. Several AQPs are inhibited by mercurials via covalent modification of cysteines close to the pore entry (Borgnia et al., 1999). Hence, it came quite as a surprise when human AQP6 was found (a) to be activated by mercury ions as well as acidic pH, (b) to conduct inorganic anions, such as nitrate and chloride, and (c) not to reside in the plasma membrane but in the membranes of intracellular vesicles of acid secreting intercalated cells in the renal collecting duct where the pH drops to 5.0 or even lower (Yasui et al., 1999; Hazama et al., 2002; Ikeda et al., 2002; Beitz et al., 2006a; Yasui, 2009). AQP6, thus, represents a gated intracellular anion channel. The physiological function, however, is still not fully resolved. Co-localization with a V-type H^+^-ATPase hints a role in vesicle acidification and urinary acid secretion to maintain the pH homeostasis of the body. AQP6 may establish – together with an additionally present CLC-5 chloride channel – the flow of chloride into the vesicles. This should counteract any accumulation of positive charge due to proton pumping of the H^+^-ATPase (Sakamoto et al., 1999; Yasui et al., 1999). Since CLC-5 is inhibited below pH 6.5, AQP6 may take over when a higher vesicular acid load needs to be generated (Yasui et al., 1999).

**FIGURE 2:**
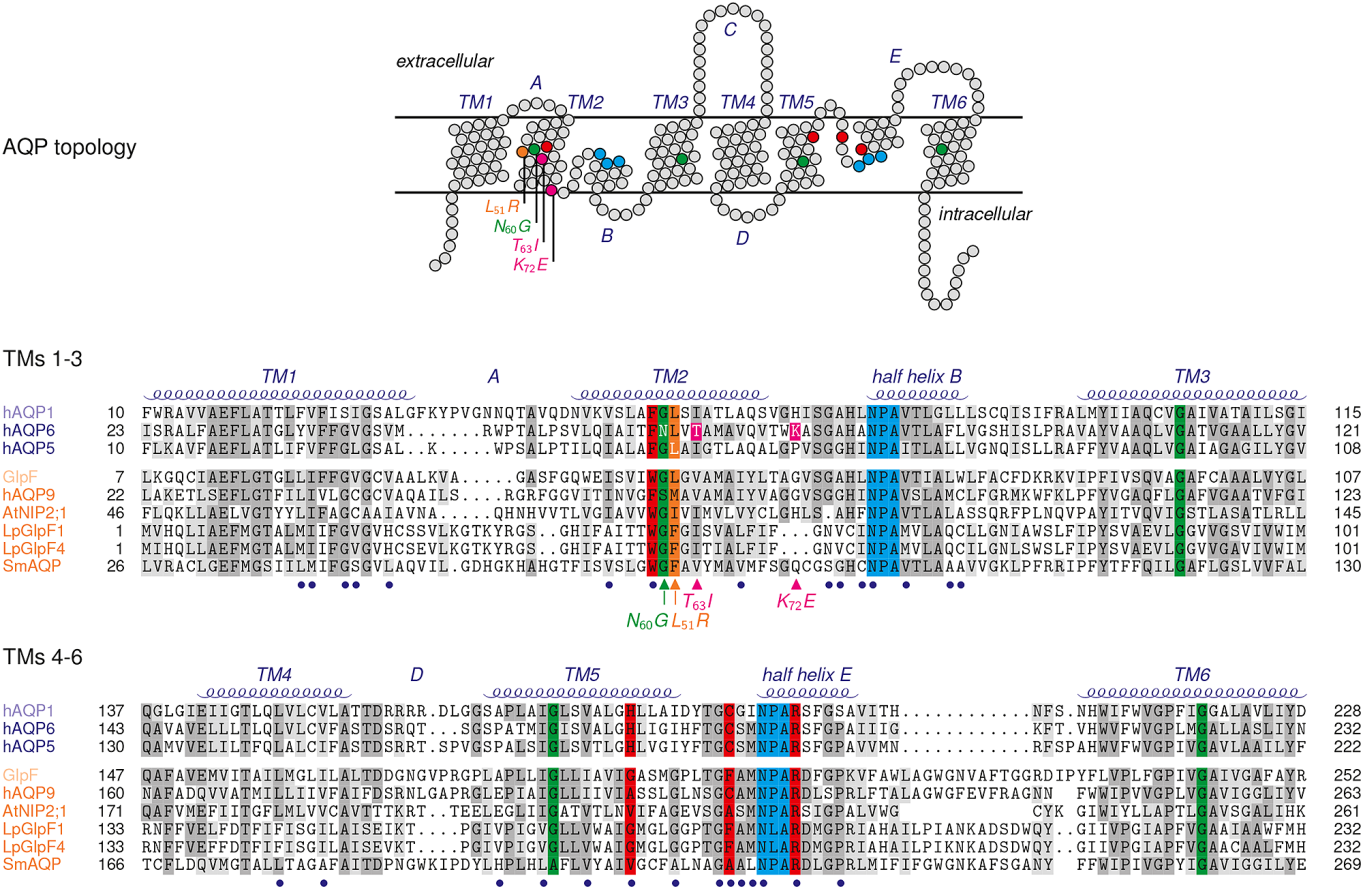
**Topology and sequence comparison of selected AQPs.** Red shading highlights residues of the ar/R selectivity filter, blue the NPA regions. Glycines of helix contact points are colored in green, and the Leu51Arg mutation site in AQP5 in orange. The Thr63Ile and Lys72Glu mutations of AQP6 are colored magenta. Connecting loops are labeled A–E, and the transmembrane spanning helices TM1-6. The termini and the connecting loop C are excluded from the sequence alignment. Blue bullets below the sequences mark residues of the AQP channel lining. The sequences are grouped according to their subfamily attribution, i.e., the water specific type (AQP1, AQP6, AQP5) and aquaglyceroporins (*Escherichia coli* GlpF, human AQP9, NIP2;1, LpGlpF1 and LpGlpF4, SmAQP).

AQP6 has a preference for nitrate, yet halide anions are channeled as well yielding the permeability sequence: NO_3_^-^ > I^-^ ≫ Br^-^ > Cl^-^ ≫ F^-^ (Ikeda et al., 2002). The passage of anions was shown not to occur via the central pore but through the individual pores of the protomers raising the question about peculiarities in the AQP6 pore layout. Reversal of the positive charge of a lysine residue, Lys72, positioned at the cytoplasmic pore mouth by mutational replacement with a negatively charged glutamate (**Figure 2**) hardly affected anion conductance (Yasui et al., 1999). Two hydroxyl-containing residues, i.e., a tyrosine, Tyr37, and a threonine, Thr63, within the AQP6 channel path were proposed to be situated in juxtaposition to the two asparagines of the NPA motive acting as a fourfold anion coordination site. Indeed, mutation of Thr63 to isoleucine reduced nitrate permeability (Ikeda et al., 2002). Full elimination of AQP6 anion permeability and, at the same time, a gain of high water permeability was eventually achieved by replacement of an asparagine residue, Asn60, located at the junction of transmembrane helices 2 and 5 by glycine (Liu et al., 2005). Contact points of transmembrane helices in AQPs and in other membrane proteins typically contain glycines, which form dents in the interacting helix surfaces locking them in place (Murata et al., 2000). As a consequence, the transmembrane domains are less prone to slipping movements against each other leading to an overall more rigid protein structure. Rigidity is important to keep the 20 Å long AQP channel with a narrow diameter of 3–4 Å open for the efficient and continuous passage of water. It is speculated that the unique Asn60 at an AQP-typical glycine position induces exactly that degree of helix slipping in AQP6 to allow anions, such as nitrate and (partially) hydrated halides, which are consequently larger than a water molecule, to enter and pass the channel (Liu et al., 2005). A recent, serendipitous finding obtained with an AQP5 mutant seems to confirm this interpretation (Qin and Boron, 2013). AQP5 is typically found in type 1 pneumocytes and salivary glands. Actually aiming at modifying the lining of the central pore of AQP5 to study gas transport properties, a leucine, Leu51, was changed to arginine. One striking effect, however, was induction of anion permeability of the four individual pores of the AQP5-Leu51Arg mutant with a permeation sequence equal to AQP6. The mutation site is not within the channel itself but lies right next to the glycine–glycine contact point of transmembrane helices 2 and 5 of AQP5 and, thus, in the very same region as Asn60 in AQP6 (**Figure 2**).

While the AQP cation filters have been studied extensively (Murata et al., 2000; Tajkhorshid et al., 2002; de Groot and Grubmüller, 2005; Beitz et al., 2006b; Wu et al., 2009; Wree et al., 2011; Kosinska Eriksson et al., 2013), the mechanisms of preventing anions from passing AQPs has caught less attention. One computational molecular dynamics analysis on hydroxide anion permeability of the *Escherichia coli* aquaglyceroporin, GlpF, has identified the negative ends of the two AQP-typical half-helix macro dipoles pointing toward both pore entries to function as an anion repellent (Jensen et al., 2005). Further, the NPA region was suggested to form an energy barrier due to strong coordination of the anion preventing it from further conduction. The latter argument of anion coordination in the NPA regions was used in an opposite way for AQP6 in fact favoring anion permeability (Ikeda et al., 2002). Certainly, more data on the issue would be desirable. The halide permeability sequences of AQP6 and AQP5-Leu51Arg are suggestive of an anion selection mechanism based on the dehydration penalty because they correlate directly with the electronegativity of the respective anions. This indicates that the energetic cost for the removal of the hydration shell alone dictates the permeability sequence. Both AQPs are, thus, so-called non-selective anion channels (Chen et al., 2010). It is tempting to ask whether non-selective anion channels display characteristics, e.g., in the amino acid composition of the pore lining or electrostatics. Such properties may enable one to predict anion permeability of AQPs by analysis of the protein sequence.

### SLAC1, PROTOTYPE OF A NON-SELECTIVE ANION CHANNEL

Recently, the crystal structure of a non-selective anion channel, i.e., the slow anion channel 1, SLAC1, has been elucidated (Chen et al., 2010). SLAC1 is found in the aperture-defining guard cells of plant stomata where it regulates the exchange of water vapor and gasses derived by photosynthesis. SLAC1 is a symmetrical trimer with each monomer comprising ten transmembrane helices. Five of these helices build an inner core forming the actual anion channel whereas the remaining five helices surround the core as an outer hull. The channel lumen has a comparatively uniform diameter of about 5 Å with one constriction site at the center where it narrows to 2 Å (closed state). Here, a highly conserved phenylalanine residue is thought to be responsible for channel gating maintaining the vital integrity of transmembrane ion gradients, a feature shared by all ion channels (pore-forming toxins excluded). The phenylalanine gate is switched open indirectly via phosphorylation of SLAC1 and subsequent conformational changes of the protein.

Somewhat counterintuitively, the SLAC1 channel is lined by mostly hydrophobic amino acid residues with a rare occurrence of hydroxyl-donating residues (16%), e.g., serine and threonine. Charged residues are absent. Nevertheless, the electrostatic potential of the cytoplasmic and extracellular protein surfaces is electropositive as derived by lysine and arginine residues located in the extra-membranous loops. This feature apparently attracts anions to the channel entry sites and simultaneously repulses cations.

The permeability sequence of SLAC1 (I^-^ > NO_3_^-^ > Br^-^ > Cl^-^ > SO_3_^2-^ > malate) inversely reflects the dehydration energies of the anions and identifies the protein as a non-selective anion channel. Anion dehydration appears to be the only selective element of SLAC1 and, accordingly, the protein structure lacks any specific anion-binding element. In general, anion channels are less specific than cation channels but a certain degree of selectivity can be achieved by proper arrangement of anion coordinating amino acids and the electrostatic landscape (Dutzler et al., 2002). The low but measurable permeability of SLAC1 for malate is notable because it may indicate that the protein undergoes conformational fluctuations incidentally widening the diameter of the channel to accommodate the bulky shape of the substrate.

Together, the layout of a non-selective anion channel appears mainly lipophilic with a few hydroxyl-providing residues interspersed and structural or compositional features that establish a positive electrostatic field to attract anions to the entry sites. Such properties can manifest themselves in a rather inconspicuous protein sequence and, thus, may be hard to detect in a protein alignment. In fact, virtually all AQPs exhibit a mixed hydrophobic/hydrophilic pore lining plus positive electrostatics (**Figure 2** and **Table 1**; Hub and de Groot, 2008). Yet, the key for inorganic anion permeability of AQPs seems to reside in the loosened pairing of transmembrane helices 2 and 5 brought about by a residue larger than glycine at the helix contact point.

**Table 1:** Pore lining amino residues of selected AQPs.

Region	hAQP1	hAQP6	hAQP5	GlpF	hAQP9	AtNIP2;1	LpGlpF1	LpGlpF4	SmAQP
TM1	F	Y	F	L	L	L	M	M	L
	V	V	V	I	I	I	I	I	M
	S	G	G	G	G	G	G	G	G
	I	V	L	V	C	C	V	V	S
	A	V	A	V	V	I	H	H	L

TM2	V	I	I	I	I	I	A	A	V
	**F**	**F**	**F**	**W**	**F**	**W**	**W**	**W**	**W**
	I	T	I	V	V	I	I	I	V
	A	V	A	I	I	V	L	L	V

Half helix B	G	G	G	G	G	S	N	N	S
	A	A	G	A	G	A	V	V	G
	L	A	I	L	I	F	I	I	C
	*N*	*N*	*N*	*N*	*N*	*N*	*N*	*N*	*N*
	V	V	I	V	V	V	M	M	V

TM4	L	L	L	L	L	L	F	F	L
	V	V	I	I	V	V	I	I	A

TM5	A	P	P	A	E	E	V	V	H
	I	I	I	I	I	I	V	V	L
	V	V	V	I	I	V	V	V	V
	**H**	**H**	**H**	**G**	**A**	**V**	**G**	**G**	**V**
	I	I	I	G	G	G	G	G	A

Half helix E	G	G	G	G	G	G	G	G	G
	**C**	**C**	**C**	**F**	**C**	**A**	**F**	**F**	**A**
	G	S	S	A	A	S	A	A	A
	I	M	M	M	M	M	M	M	L
	*N*	*N*	*N*	*N*	*N*	*N*	*N*	*N*	*N*
	**R**	**R**	**R**	**R**	**R**	**R**	**R**	**R**	**R**
	S	P	P	P	P	P	P	P	P

Positive (H,R)	2	2	2	1	1	1	2	2	2
Polar (S,T,Y,N)	3	5	3	2	2	4	3	3	4

*Residues of the constriction sites are highlighted (ar/R region: bold, upright characters; NPA region: slanted letters).*

**FIGURE 3:**
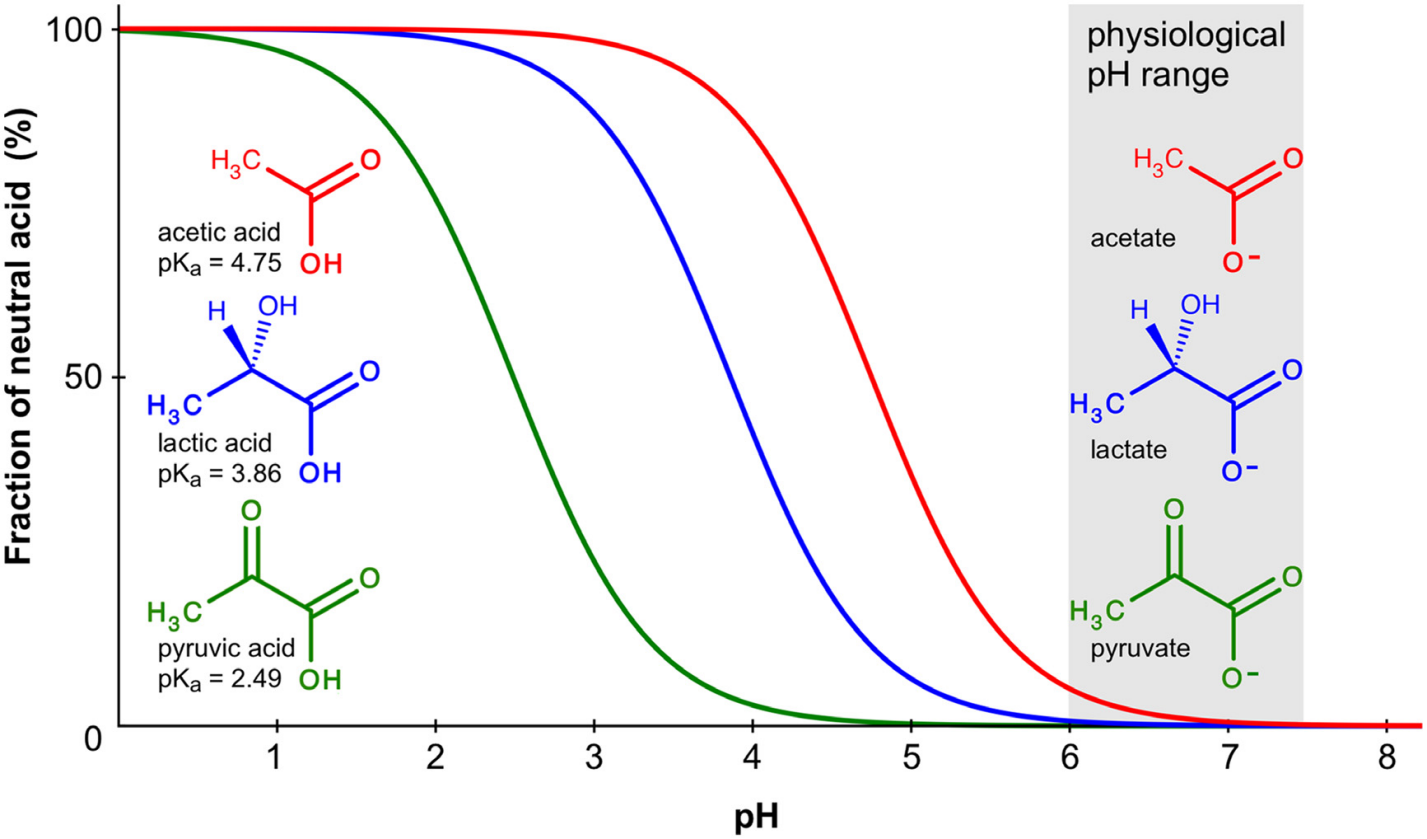
**Chemical protonation equilibrium of weak monoacids.** The curves indicate the fraction of the protonated, neutral forms of acetic, lactic, and pyruvic acid in a pH range from 0 to 8. Calculations were done using the Hendersen–Hasselbalch equation; p*K*_a_ values are given at the molecule structures.

Besides inorganic anions, a physiological environment contains organic metabolites with negative charges in the form of carboxylates, such as acetate, pyruvate, or lactate. Here, the situation is complicated by the pH-dependent chemical protonation equilibrium, which renders the compounds anionic in the neutral and alkaline range and uncharged in the acidic range (**Figure 3**). Several reports have suggested permeability of certain AQPs for monocarboxylates (Tsukaguchi et al., 1998; Choi and Roberts, 2007; Faghiri et al., 2010; Bienert et al., 2013).

## AQPs WITH PERMEABILITY FOR ORGANIC MONOACIDS OR MONOCARBOXYLATES

### HUMAN AQP9 FACILITATES LACTIC ACID AND 2-HYDROXYBUTYRIC ACID

The first AQP that was associated to monocarboxylate permeability was human AQP9, an aquaglyceroporin predominantly expressed in liver, testis and brain (Tsukaguchi et al., 1998). AQP9 was shown to conduct a variety of solutes including urea, polyols, and monocarboxylates. The initial report suggests that even larger molecules containing bicyclic purine moieties can pass the AQP9 channel (Tsukaguchi et al., 1998). AQP9 was not shown to conduct inorganic anions. A reasonable degree of permeability for monocarboxylates, such as lactate, would link respective AQPs to energy metabolism. In fact, it is speculated that AQP9 is involved in the transport of lactate, and ketone bodies including the acidic 2-hydroxybutyrate across the blood-brain barrier (Tsukaguchi et al., 1998; Badaut et al., 2004). The physiological relevance must be questioned, however, in the light of specialized monocarboxylate transporters (MCTs) of which 14 genes have been identified in the human genome (Halestrap, 2013). MCTs are classical secondary transporters that make use of proton or sodium gradients and that can be described by the alternate access model. They possess specific binding sites for the substrate, e.g., lactate, and a co-substrate, e.g., a proton, and undergo a major conformational shift subsequent to the binding step enabling release of the substrate and co-substrate at the other side of the membrane.

How would AQP monocarboxylate permeability fit into a physiological setting and what is the mode of conduction? At physiological pH, the monocarboxylate anion by far outweights the protonated, neutral acid form (**Figure 3**). Lactic acid, for instance, has a p*K*_a_ of 3.9 resulting at pH 7.4 in a molar ratio of 99.97% lactate anions vs. 0.03% neutral acid molecules. Acidification will increase the fraction of the neutral form 10-fold with each pH unit. Accordingly, when conducting functional permeability assays with monocarboxylates, it is necessary to carefully control the pH of the test buffer because it may directly affect the outcome of the experiment due to the chemical protonation equilibrium.

Likewise, *Xenopus laevis* oocytes expressing AQP9 exhibited a fourfold increase in lactate permeability when the assay pH was shifted from 7.4 to 5.5 (Tsukaguchi et al., 1998). Two mechanistic principles can be envisioned to explain the pH-dependency. (a) AQP9 may acts as a lactate/proton co-transporter using an energizing transmembrane proton gradient, and (b) AQP9 acts as a channel for the protonated lactic acid form. The second scenario clearly appears favorable taking into account the typical channel structure of AQPs. This view is supported by the fact that AQP9 conducts 2-hydroxyburyrate three times faster than lactate despite its larger size by one additional methylene unit in the molecule chain (Tsukaguchi et al., 1998). The crucial difference to lactate may not be the increased chain length of 2-hydroxybutyrate but its higher p*K*_a_ of 4.7. This translates into a weaker tendency to dissociate resulting in 6.3 times higher concentrations of the neutral 2-hydroxybutyric acid form. The respectively elevated transmembrane gradient of 2-hydroxybutyrate explains the higher permeability rate very well. The matter could be finally resolved by carrying out AQP9 monocarboxylate permeability assays in the presence of proton gradient uncouplers, e.g., the protonophore carbonyl cyanide m-chlorophenyl hydrazone (CCCP; McLaughlin and Dilger, 1980). Under these conditions, a proton co-transport mechanism will cease whereas the channeling rate of protonated monoacids will remain unaltered.

The protein sequence of human AQP9 displays highest similarity to human AQP7 (48% identity, 85% similarity) for which no anion permeability has been shown (Ishibashi et al., 1997). There are no peculiarities in the amino acid composition of the constriction sites and filter regions of AQP9. However, the highly conserved glycine contact site in transmembrane domain 2 holds a larger serine residue, Ser65, i.e., the position where AQP6 carries the untypical Asn60 (Liu et al., 2005). One may speculate that, analogous to Asn60 in AQP6, Ser65 could disturb the helix contact with transmembrane domain 5 loosening rigidity within the AQP9 structure and allowing larger molecules, e.g., the observed purines, to pass. A confirming high-resolution crystal structure of AQP9 is not yet available, but a 7 Å resolution projection map obtained from electron microscopy of 2D crystals indeed indicates a wider selectivity filter region compared to other aquaglyceroporins (Viadiu et al., 2007).

### A PLANT AQP CONDUCTS PROTONATED, NEUTRAL LACTIC ACID

A member of the nodulin 26 intrinsic proteins (NIPs), i.e., a plant specific AQP subfamily, has been shown to be permeable for lactic acid (Choi and Roberts, 2007). NIPs typically conduct water and small uncharged solutes, such as glycerol (Wallace et al., 2006). NIP2;1 from *Arabidopsis thaliana*, however, shows minimal water and glycerol facilitation (Choi and Roberts, 2007). It is highly expressed in root tips under anaerobic conditions, i.e., waterlogging. When expressed in *Xenopus* oocytes, a pH dependent permeability for lactic acid was observed in a range from neutral to an acidic pH of 3.5. The uptake of lactic acid was clearly noticeable only below pH 5 and the permeability rate increased exponentially with further increasing acidity, thus, perfectly mirroring the proportion of neutral lactic acid in the assay buffer solution. Importantly, the most acidic condition tested was beyond the p*K*_a_ of lactic acid, i.e., a point at which the fraction of lactate anions is very low. Still, the uptake rate increased, indicating that not the anion but the neutral lactic acid form binds to and passes the NIP2;1, and disfavoring a monocarboxylate/proton co-transport mechanism (Choi and Roberts, 2007).

There are examples in the literature displaying substantial permeability of bacterial and parasite AQPs for lactate or lactic acid even at neutral pH, i.e., when the proportion of the neutral acid is low (Faghiri et al., 2010; Bienert et al., 2013). Other than in humans, which express specialized MCTs in addition to AQPs, in microbes, transport of monocarboxylates via AQPs may be of considerable physiological relevance.

### LACTATE PERMEABLE AQPs FROM LACTIC ACID BACTERIA

The first prokaryotic AQPs for which lactic acid permeability has been shown were identified in the genome of *Lactobacillus plantarum*, i.e., a Gram-positive lactic acid bacterium (Bienert et al., 2013). It is found in fermented food products as well as in the saliva of animals. In a phenotypic assay, growth on lactate media of a mutant *Saccharomyces cerevisiae* yeast strain lacking endogenous lactate transporters could be restored by expressing certain *L. plantarum* aquaglyceroporins, i.e., LpGlpF1 and LpGlpF4. Analyses in *Xenopus* oocytes revealed lactic acid transport rates for LpGlpF4 similar to AQP9. Permeability was further indirectly assessed using *L. plantarum* bacteria at pH 7.5/6.5/5.5, which exhibited a pH dependency of the whole-cell lactate racemization activity, an assay used to estimate the transport of lactic acid across cell membranes. This led the authors to propose that lactic acid constitutes the conducted molecule and not the lactate anion, fitting the picture generated from the eukaryotic AQPs discussed above. *L. plantarum* expresses three more aquaglyceroporins for which no lactic acid permeability was observed. Direct comparison of the ar/R selectivity filter of LpGlpF1-6 yielded the same amino acid composition, i.e., it is not decisive for lactic acid selectivity (Bienert et al., 2013). An exclusive feature of the *L. plantarum* lactic acid facilitating LpGlpF4 might be a methionine following the first NPA motive, this position holds a valine in the other *L. plantarum* aquaglyceroporins. It has been shown before, that the layout of the AQP channel lining directly affects solute selectivity and contributes to discrimination between different diastereomeric forms of polyols (Beitz et al., 2004).

### A PARASITIC NEMATODE AQP AS A LACTIC ACID FACILITATOR IN ENERGY METABOLISM

The pathogenic trematode *Schistosoma mansoni* is transmitted between various hosts including water snails and humans, and undergoes a complex developmental life cycle (Faghiri et al., 2010). The main energy generating biochemical pathway is glycolysis continuously producing lactic acid that needs to be removed from the organism to keep it viable. MCTs of the MCT family appear to be absent from *Schistosoma mansoni* bringing up the question as to how the parasite accomplishes lactic acid removal (Skelly and Alan Wilson, 2006; Faghiri et al., 2010). The identification of the lactate facilitating protein is expected to have relevance as a novel drug target. Recently, an aquaglyceroporin of the parasite, SmAQP, was shown to conduct lactic acid even at neutral pH (Faghiri et al., 2010). Permeability increased about fourfold when the pH was shifted from 7.4 to 6.3. Using *Xenopus* oocytes a low affinity *K*_M_ value of 41 mM and an activation energy of 7.18 kcal mol^-1^ was determined consistent with facilitation via a channel protein rather than a transporter.

Although these findings again fit very well into the scheme of AQPs conducting the protonated form of lactic acid it is notable that at neutral pH with an overweight of the anion over the neutral acid by three orders of magnitude there is still an apparent and probably physiological relevant level of transport via SmAQP (**Table 2**).

**Table 2:** Assay conditions and observed increase of AQP facilitated lactate transport.

	Ext. pH	Lactate gradient (mmol l^-1^)	Neutral acid fraction (%)	Lactate transport via membrane (pmol min^-1^ oocyte^-1^)	Lactate transport via AQP (pmol min^-1^ oocyte^-1^)	Ratio of transport AQP/membrane
AQP9	7.4	1	0.03	9.8	13.1	1.3
	5.5	1	2.45	?	51.1	?

NIP2;1	7.6	20	0.02	27.0	26.0	1.0
	6.0	20	0.79	43.0	43.0	1.0
	5.0	20	7.36	27.0	35.0	1.3
	4.5	20	20.08	76.0	108.0	1.6
	4.0	20	44.27	85.0	227.0	2.7
	3.5	20	71.53	139.0	521.0	3.7

LpGlpF1	7.0	80	0.08	45.3	62.2	1.4

LpGlpF4	7.0	80	0.08	45.3	91.5	**2.0**

SmAQP	7.4	1	0.03	7.2	25.2	**3.5**
	6.3	1	0.36	?	54.3	?

*Compared are transport rates via the *Xenopus laevis* oocyte plasma membrane and via AQP9 (Tsukaguchi et al., 1998), NIP2;1 (Choi and Roberts, 2007), LpGlpF1/LpGlpF4 (Bienert et al., 2013), and SmAQP (Faghiri et al., 2010). Bold numbers indicate AQP/membrane transport ratios ≥ 2 at neutral pH.*

### COMPARISON TO MONOCARBOXYLATE TRANSPORT VIA FORMATE NITRITE TRANSPORTERS (FNTs)

Recently, the protein structures of various members of a microbial MCT family, i.e., formate nitrite transporters (FNT; Suppman and Sawers, 1994), have been elucidated, which are related to AQPs in several striking ways (Wang et al., 2009; Waight et al., 2010; Lü et al., 2011, 2012; Czyzewski and Wang, 2012). FNTs are found exclusively in lower organisms, such as bacteria, archaea, fungi, algae, and unicellular parasites, and transport inorganic anions (nitrite; Lü et al., 2012) and monocarboxylates (formate, acetate, pyruvate, lactate; Lü et al., 2013). The proteins assemble to symmetric homopentamers with each protomer forming an individual pore. The central domain of the pentamer has not been linked to transport function, yet. Each protomer consists of six transmembrane helices and two half helices and closely resembles the aquaporin topology. It is even possible to overlay FNT and AQP structures resulting in minimal deviations in the Angstrom range of the protein backbones, a phenomenon referred to as molecular mimicry (Wang et al., 2009). However, there is no homology in the FNT and AQP protein sequences. Still, FNTs and AQPs share even more structural details: the FNT transduction pore contains two constriction sites enclosing a hydrophobic channel section. The channel diameter is about 3.5 Å and 1.8 Å at the constriction sites indicating that conformational changes are required for accommodating monocarboxylates as substrates.

The outer constriction, located toward to periplasmic space in bacteria, harbors a highly conserved histidine, which has been shown to be critical for FNT anion facilitation (Waight et al., 2010). Two functions can be attributed to the histidine residue. First, in its protonated form, the histidine electrostatically attracts anions to the channel entry. Second, the proton can be transferred to the anion substrate converting it into the neutral acid form that is compatible with the hydrophobic channel interior. The whereabouts of the transferred proton after the release of the substrate from the channel are still under debate and may be different for individual FNTs (Lü et al., 2013). Further, the prevailing pH conditions are discussed to affect the fate of the proton (Lü et al., 2012). Two scenarios are immanent. Either the proton remains with the substrate resulting in a monocarboxylate/proton co-transport mechanism, or the FNT releases the substrate anion due to re-abstraction of proton, which finds its way back to the protein and eventually re-protonates the histidine. Experimental data and the crystal structures point toward the latter mechanism as the prevailing one (Lü et al., 2012). The FNT channel lining contains, besides the conserved histidine of the periplasmic constriction, an equally conserved threonine. Its hydroxyl moiety is involved in the coordination of a water molecule that is permanently fixed to the protein. It is the current view that the histidine/threonine/fixed water triad acts as a bidirectional proton relay that transiently protonates the substrate and recycles the proton after substrate release. By addressing the chemical properties of the substrate, i.e., the p*K*_a_ of a weak acid, the proton relay constitutes a crucial part of the channel selectivity mechanism.

At acidic conditions at one side of the membrane a proton motive force builds up, which may energize monocarboxylate/proton co-transport via FNTs. In fact, there is evidence that at pH 5.7 a bacterial FNT, FocA, switches to secondary, proton-driven monocarboxylate transport (Lü et al., 2011, 2012). A drop of the cytosolic pH occurs for instance during anaerobic energy metabolism involving fermentation of various weak monoacids, such as lactate, i.e., a physiological condition for which the AQPs from *Lactobacillus* and *Schistosoma* spp. have been connected to monocarboxylate transport as well (Faghiri et al., 2010).

### RELEVANCE OF AQPs IN MONOCARBOXYLATE TRANSPORT

At least three protein families seem to be involved in transmembrane monocarboxylate transport. These are classical secondary transporters of the MCT type, the more channel-like, microbial FNT proteins, both representing specialized MCT families, and the AQPs. MCTs and FNTs have evolved mechanisms of attraction, selectivity and transduction for monocarboxylates whereas AQPs appear to be mainly passive elements. Yet, certain AQPs increase the lactate permeability of plasma membranes even in the neutral pH range (**Table 2**), where the neutral lactic acid form is underrepresented by three orders of magnitude. It is thinkable that the positive electrostatic field of AQPs derived from the half-helix dipole moment and the positive charge in the ar/R region draws lactate anions to the pore entries increasing the local concentration of the substrate. Due to the chemical equilibrium and depending on the pH a certain fraction of lactate anions will be protonated by the medium to convert to neutral lactic acid molecules, which can pass the AQP channel following the concentration gradient. Hence, two properties of an AQP should affect its permeability for monocarboxylates, the strength of the electrostatic field, and the compatibility of the channel lining with the passing monoacid in terms of diameter and interaction sites, mainly hydrogen bond donor and acceptor sites. Unfortunately, systematic experimental analyses are missing.

Monocarboxylate transporters and FNTs appear much better suited for transporting monocarboxylate across lipid membranes. Yet, in the absence of such transporters and at appropriate physiological conditions, AQPs should be able to carry a considerable load of monocarboxylates, especially along transmembrane pH gradients.

### SUITABILITY OF AQPs FROM HUMAN PATHOGENS AS DRUG TARGETS OR VEHICLES FOR DRUG UPTAKE

There are documented examples of phenotypes resulting from knockdown, deletion, or overexpression of AQPs in parasites, which expose these AQPs as putative therapeutic targets. In selected cases AQPs even serve as uptake pathways for certain drug molecules (recently reviewed by Song et al., 2014).

For instance, expression levels of the *Schistosoma* SmAQP have been reduced by RNA interference experiments in culture (Faghiri et al., 2010). The knockdown severely affected the juvenile schistosomula form of the parasites, which appeared stunned and only a small number of parasites survived in culture. The authors attribute this behavior to impaired osmoregulation due to the loss of SmAQP. Adult parasites survived the knockdown of SmAQP very well but exhibited a strongly reduced rate of lactic acid secretion.

Another example deals with the malaria parasite. Here, the knockout of the single AQP gene in the rodent *Plasmodium berghei* strain reduced the uptake of glycerol, a precursor of glycerolipids, from the host and slowed the growth rate to about half (Promeneur et al., 2007). As a result, mice infected with the AQP-knockout strain carried less parasites in their red blood cells and survived twice as long as those infected with wild-type parasites. The central role of AQPs from both, the host and the *Plasmodium* parasite, as constituents of the interface for glycerol exchange has been further shown in knockout mice lacking AQP9. Red blood cell AQP9 is the mouse counterpart to the *Plasmodium* AQP and the lack of AQP9 equally reduced glycerol uptake and the virulence of plasmodia (Liu et al., 2007).

The cases described above highlight physiological functions of parasite AQPs in the exchange of metabolites with the host. Therapeutic interference with the interfacing AQPs may cut off the parasites from biosynthetic precursor molecules or may lead to intracellular accumulation of waste products. Either should negatively affect development and probably virulence. Accordingly, such parasite AQPs are considered putative drug targets.

Other reports have shown that parasite AQPs can be used as uptake routes for anti-parasitic drugs, e.g., in the case of Leishmaniasis, which is still treated with pentavalent arsenic or antimony containing drugs. The compounds release by chemical reduction arsenous, As(OH)_3_, and antimonous acid, Sb(OH)_3_, respectively. Both are very weak acids with p*K*_a_ values of 9.2 and 11.8 meaning that they hardly deprotonate at neutral pH. The drug products enter *Leishmania major* parasites as uncharged molecules via LmAQP1 (Gourbal et al., 2004; Marquis et al., 2005).

A striking and highly unexpected drug uptake mechanism has been identified in *Trypanosoma brucei*, i.e., the causative agent of human African trypanosomiasis, also kown as sleeping sickness. Here, an aquaglyceroporin, TbAQP2, is unequivocally connected to the high-affinity transport of a quite large (340 Da) and double positively charged drug molecule, pentamidine (Alsford et al., 2012; Munday et al., 2014). The TbAQP2 selectivity filter is somewhat unusual in that it lacks the highly conserved arginine residue but carries a leucine instead (Uzcategui et al., 2004). Further, both NPA motifs are changed to NSA/NPS. Whether these structural peculiarity enable TbAQP2 to conduct pentamidine directly via the AQP pore or whether additional proteins are involved in the process is not yet finally resolved (Baker et al., 2012; Munday et al., 2014). Anyhow, the uptake of pentamidine demonstrates that AQPs can deliver even larger drug molecules to human-pathogenic parasites opening up as yet unforeseen therapeutic avenues.

## CONCLUSION

Permeability of AQP6 for inorganic anions is unique but appears real. The conversion of AQP5 into an anion channel by a single point mutation shows that the AQP pore layout is generally compatible with anion conductivity. Yet, the AQP structure does not carry a specialized domain for energy efficient removal of the anion hydration shell. Only loosening of the protein’s transmembrane rigidity leading to thermal fluctuations of the pore diameter allows partially hydrated anions to pass in a sequence determined by the dehydration penalty.

With regard to permeability of AQPs for monocarboxylates more complete data sets would be appreciable. The current data, however, strongly hint at the passage of the protonated, neutral acid form. Still, various AQPs may be better suited to attract and conduct weak monoacids, such as lactate, depending on the strength of the positive electrostatic field, their pore diameter, and compatibility with the stereochemical configuration of the substrate by providing properly oriented coordination sites within the pore. Contrary to FNTs, the agent for substrate protonation appears not to be a particular residue of the AQP but hydronium ions from the medium. Hence, conditions favoring monocarboxylate permeability of AQPs are an increased local substrate concentration in combination with a low pH. This situation typically arises in microbes during anaerobic energy metabolism with lactate and protons being the end products of glycolysis. Accordingly, AQPs could contribute, or, in the absence of specialized transporters, may be even solely responsible for lactate release from a cell. Such a context in a human pathogen would render the respective AQP an attractive drug target.

## Conflict of Interest Statement

The authors declare that the research was conducted in the absence of any commercial or financial relationships that could be construed as a potential conflict of interest.
